# Synthesis, band gap structure and third order non-linear optical properties of zinc tungsten oxide nanocomposite using a single CW laser beam

**DOI:** 10.3389/fchem.2023.1152501

**Published:** 2023-04-14

**Authors:** Zahra Jalili, Ehsan Koushki, Amir Hossein Ehsanian, Reza Tayebee, Behrooz Maleki

**Affiliations:** ^1^ Department of Chemistry, Faculty of Sciences, Hakim Sabzevari University, Sabzevar, Iran; ^2^ Department of Physics, Faculty of Sciences, Hakim Sabzevari University, Sabzevar, Iran; ^3^ Department of Organic Chemistry, Faculty of Chemistry, University of Mazandaran, Babolsar, Iran

**Keywords:** zinc tungsten oxide, Z-scan measurement, saturation absorption, thermal lensing, third order non-linear optical

## Abstract

In this study, a composite of zinc tungsten oxide nanoparticles (W-ZnO NPs) has been synthesized via mixing Na_2_WO_4_ and zinc acetate in water, followed by dropwise addition of NaOH. The synthesized W-ZnO NPs were characterized using measurement methods such as XRD, dynamic light scattering (DLS), Scanning electron microscopy (SEM) and UV-Vis. Also, the results were compared with the pure synthesized ZnO and WO_3_ NPs. Non-linear optical properties of the synthesized composite were measured using the Z-scan technique with a continuous wave Nd-Yag laser. The negative non-linear absorption coefficient of the components was obtained which indicates that the saturation absorption occurred in this composite. In comparison with pure ZnO NPs, non-linear absorption decreases which can be attributed to the negligible optical response of WO_3_ structures. Also, the negative value of the close aperture Z-scan curve shows that the thermal lensing effect is the main reason for the third-order non-linear refraction.

## 1 Introduction

Recently, the use of inorganic materials in electronic and optical devices as active elements, such as light-emitting diodes, photovoltaic devices, and field-effect transistors has received much attention from the outlook of potential technological applications as well as fundamental science (Cao et al., 2011; Akherat Doost et al., 2021; Ashfaq et al., 2022). Meanwhile, metal oxide NPs (MONPs) are more important in technology because of their unique semiconducting properties (Silva et al., 2019) and play a considerable role in a multitude of fields of chemistry, physics and material sciences. MONPs are commonly known as catalysts, gas sensors, absorbents, superconductors, semiconductors and ceramics (Naseem and Durrani, 2021). Due to the effect of morphology and size on the properties of metal oxide NPs, their synthesis techniques mainly focus on these two parameters (Maduraiveeran et al., 2019). Among MONPs, the transition metal oxides have drawn much notice due to their outer electron configuration (Velmurugan and Incharoensakdi, 2018)

Among various classes of metal oxide NPs, II-VI class inorganic semiconductor nanomaterials like Zinc Oxide (ZnO), Cadmium Sulfide (CdS) and Zinc Sulfide (ZnS) have emerged as important materials for the applications in photovoltaic and optoelectronics devices (Abbasi et al., 2017). High chemical stability, inexpensive and easy synthesis methods and fast optical response speed lead to the use of them in non-linear optical devices such as optical switchers (Koushki et al., 2014a).

Zinc oxide (ZnO) and tungsten oxide (WO_3_) are among the most widely used transition metal oxides. ZnO is a semiconductor with a wide band gap, good electrical conductivity and high transmittance (Nakahara et al., 2001). ZnO is one of the most highly used materials in catalysts and photocatalysts, optical materials, solar cells, UV absorbers in cosmetics, biosensors and nanogenerators (Gao et al., 2005; Srivastava et al., 2013). In addition to the above features, ZnO NPs have the potential for applications as non-linear optical devices, optical switchers, and have been studied extensively (Baedi et al., 2021). Being a member of group II-VI compounds, ZnO NPs have been extensively used in photovoltaic and optoelectronic devices. On the other hand, WO_3_ NPs also have far-reaching usages in various fields such as batteries, gas sensors, catalysts and photocatalysts, illumination and electronics (Widenkvist et al., 2008; Hariharan et al., 2019). The synthesis of ZnO NPs doped WO_3_ has also been investigated in a few articles, from the viewpoint of structure and photocatalytic properties (Arshad et al., 2020).

In the present work, WO_3_, ZnO and zinc tungsten oxide NPs (W-ZnO NPs) have been synthesized through hydrothermal and chemical methods, and characterized using some measurement methods such as XRD, DLS, SEM and UV-Vis. After the evaluation of the band gap structure, the third-order non-linear optical properties of the synthesized composite were measured using the Z-scan method (Sheik-Bahae et al., 1990), and interesting results about the origin of non-linear absorption and refraction were offered at low laser irradiations regimes. Z scan technique was first discovered by Sheik-Bahae and his colleagues, and it is still used because of simplicity and highly sensitivity in measuring non-linear optical properties of materials (Sharma et al., 2012). To obtain the non-linear optical information of the material, it is enough to move the sample along the laser beam and detect the transmitting power through the sample. This experiment found that WO 3 doping can reduce the non-linear absorption coefficient and consequently the saturation of absorption of W-ZnO NPs [18] while increasing the absorption of the composite. It was directly related to the band gap structure of the synthesized composite, and discussed from the viewpoint of the physical properties of the charge carriers. The main novelty of this study is the investigation of tungsten dopant effects on the optical properties, bandgap and subsequently non-linear optical properties of ZnO nanoparticles.

## 2 Experiments

### 2.1 Experimental methods

The synthesized nanostructures were characterized by X-ray diffraction (XRD, DMAX2500, Rigaku X-ray diffractometer) in the 2θ domain at 30 mA and 40 keV and a scanning rate of 3° min^-1^ from 10° to 80° with Cu Kα radiation. The morphology and size of NPs were studied using a scanning electron microscope (SEM, MIRA3, TESCAN, Czech Republic) and a DynaPro NanoStar dynamic light scattering (DLS) detector (WYATT Technology). UV-visible spectra were recorded with a EU_2200 spectrophotometer.

**FIGURE 1:**
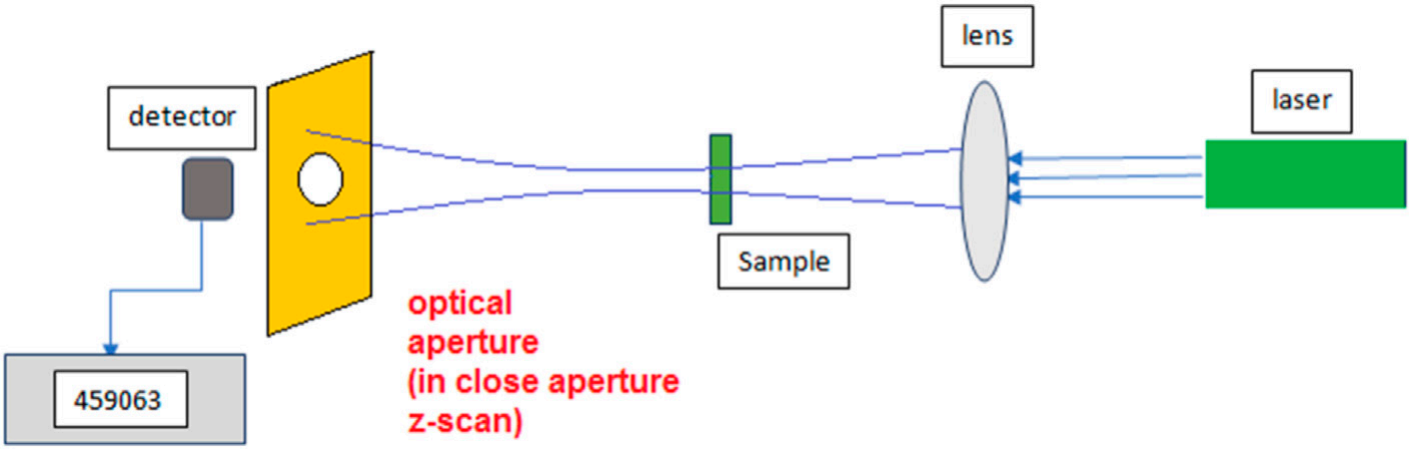
Schematic of Z-scan technique.

Z scan setup contains a continuing wave (CW) Nd-Yag laser, a convergence lens, and a detector. The laser beam, which has a Gaussian distribution, propagates along the *Z* direction. The lens is placed perpendicular to the *Z* direction. After the lens, the laser beam is focused, and the sample containing the nano-colloid scans the *z*-axis before and after the focus. The beam falls completely into the detector to measure the transmitted power. In a close aperture setup which is due to the non-linear refraction, a finite aperture is placed before the detector, as shown in Figure 1.

The wavelength of the used green laser in close and open aperture Z-scan curves was 532.8 nm (CNIlaser/MSL-S-532-S). The output power of the laser was about 5mW, and the beam was focused using a convergence lens with a focal length of 8 cm. The beam waist at the focal point was measured at about $w_{0}=35\mu m$ using the well-known edge-scan technique (Suzaki and Tachibana, 1975), and the power after focusing on the lens was 4.35 mW. Also, the Rayleigh range and the on-axis peak intensity were obtained $z_{0}=7.2mm$ and I0=228Wcm2 , respectively. Transmitted powers were measured using a semiconductor photo-detector (Ashabeam/PMB-101/IRAN). Colloidal solutions of NPs with a concentration of 0.01M in water have been prepared and poured into a 1 mm thickness quartz cell that was used as an optical sample. In a close aperture setup, a circular aperture with a radius of $r_{a}=0.2mm$ was used.

## 3 Theory of Z-scan method

In the presence of the laser beam, the absorption coefficient (*β*) and also the refractive index ( $n_{2}$ ) of non-linear materials would change according to (Sheik Bahae et al., 1990; Tsigaridas et al., 2003):

$$n=n_{0}+n_{2}I\;and\;\alpha =\alpha_{0}+\beta I$$ (1)

where $n_{0}$ and $\alpha_{0}$ are refractive index and absorption coefficient in low intensities, respectively. In the close aperture Z-scan experiment, the sample scans the *z*-axes (parallel to beam direction) before and after the focal point and, the transmitted power through an optical aperture (radius of $r_{a}$ ) is plotted via the sample position (*z*). It gives rise to the close aperture Z-scan curve, and by curve fitting, experimental curves with theoretical one, $n_{2}$ would be obtained. The focal point is considered the origin of the *z*-axis (*z* = 0). The far-field at the aperture plane (*z + D*) is given by (Sheik Bahae et al., 1990; Koushki and Majles Ara, 2011):

Er,z+D=Einr=0,ze−αL2∑m=0∞−iΔφz,r=0mm!wm0wmexp−r2wm2−ikr22Rm+iθm (2)

In this relation, *D* is the distance between the aperture and the sample, and $E_{in}\left(r=0,z\right)$ is the electric field of the incident beam on the sample plane. We also have *r* as the radial coordinates, *K,* the wave number, $\Delta \varphi \left(z,r=0\right)$ , the phase change of the beam at the center of the beam, and *L,* the thickness of the sample. Now, we will have the optical phase change as follows:

$$\Delta \varphi \left(z,r\right)=\frac{kn_{2}I_{0}L_{eff}}{1+{\left(\frac{z}{z_{0}}\right)}^{2}}\exp\left(-2r^{2}/w^{2}\left(z\right)\right)$$ (3)

and also Leff=1−e−αLα , $z_{0}$ and $I_{0}$ are the effective length of the sample, the Rayleigh length and the intensity at the center of the beam waist, respectively. By defining g=1+DRz , we will have other terms in Eq. 2 as below:

wm02=w2z2m+1,dm=kwm022,wm2=wm02g2+D2dm2,Rm=D1−gg2+D2dm2−1

θm=tan−1Ddmg (4)

where $w\left(z\right)$ is the radius of the beam at z position, we can obtain the transmitted power, which obeys the following relation:

$$P\left(z\right)=\int_{0}^{r_{a}}I\left(r,z\right)2\pi rdr$$ (5)

So, at any point of z, the normalized transmittance follows:

$$T\left(z\right)=\frac{\int_{0}^{\infty }P\left(z,\Delta \varphi_{0}\left(t\right)\right)dt}{\int_{0}^{\infty }P\left(z,\Delta \varphi_{0}=0\right)dt}$$ (6)

Eq. 6 gives close aperture Z-scan curve which is sensitive to the non-linear refractive index and, it can be evaluated by the suitable curve fitting with experimental data $n_{2}$ . To evaluate the non-linear absorption coefficient, it is enough to match the experimental data with the theoretical curve. But when we want to perform the open aperture experiment, the aperture should be removed, and the sample should be directly exposed to the laser beam. The related relations are given as (Sheik Bahae et al., 1990);

$$P\left(z\right)=P_{0}e^{-\alpha L}\frac{\mathrm{Ln}\left(1+q_{0}\left(z\right)\right)}{q_{0}\left(z\right)}$$ (7)

where q0z=βI0Leff1+zz02 . By curve fitting, the normalized curve with Eq. 7, *β* would be obtained.

## 4 Synthesis of NPs

Here, the experimental routes for the synthesis of NPs were offered.

### 4.1 Synthesis of WO_3_

We used a reliable technique based on the recommendations of Hassani et al., 2011 to synthesize WO_3_. A mixture of Na_2_WO_4_.2H_2_O (0.4 g) and NaCl (0.15 g) was dissolved in deionized water (2 mL) under magnetic stirring. HCl (3 M) was added dropwise to the stirring solution until the solution became acidic. The solution was heated into in an autoclave at 180°C for 2 h. Being under room temperature for a time span of 1 day, the precipitate was centrifuged (2 min, 30,000 rpm) with ethanol and distilled water, and then dried at 60°C for 8 h.

### 4.2 Synthesis of ZnO

ZnO was prepared as described by Liu et al., 2014. For this purpose, a mixture of zinc acetate (Zn(AC)_2_, 0.75 g) and sodium peroxide (Na_2_O_2_, 0.4 g) was dissolved into 40 mL of deionized water under stirring. 20 min later, the mixture was centrifuged, and the separated final white product was washed with distilled water several times, and then dried at 50°C for 8 h.

### 4.3 Preparation of W-ZnO NPs

As reported in the previous work (Chen et al., 2021), W-ZnO was synthesized via mixing 2.5 mL sodium tungstate (Na_2_WO_4_.2H_2_O, 0.5 M) and 22 mL zinc acetate dihydrate (Zn(CH₃CO₂)₂·2H₂O, 1 M) in water, followed by dropwise addition of 35 mL NaOH (4 M) and stirring at room temperature (for 3 hours) to form the precursor. The suspension was then transferred into a glass conical flask (250 mL) with a ground glass stopper, and then water was added up to the marked line, and the temperature was maintained at 95 C for 10 h. The produced precipitate was collected, washed with deionized water, and dried at ambient temperature in the air.

## 5 Results and discussion

### 5.1 Characterization and structural identification of NPs

As carefully described in the experimental section, WO_3_, ZnO and W-ZnO NPs were synthesized through reliable techniques. The resulting nanomaterial was characterized by means of various techniques including XRD, DLS, ESM and UV-Vis as follows.

**FIGURE 2:**
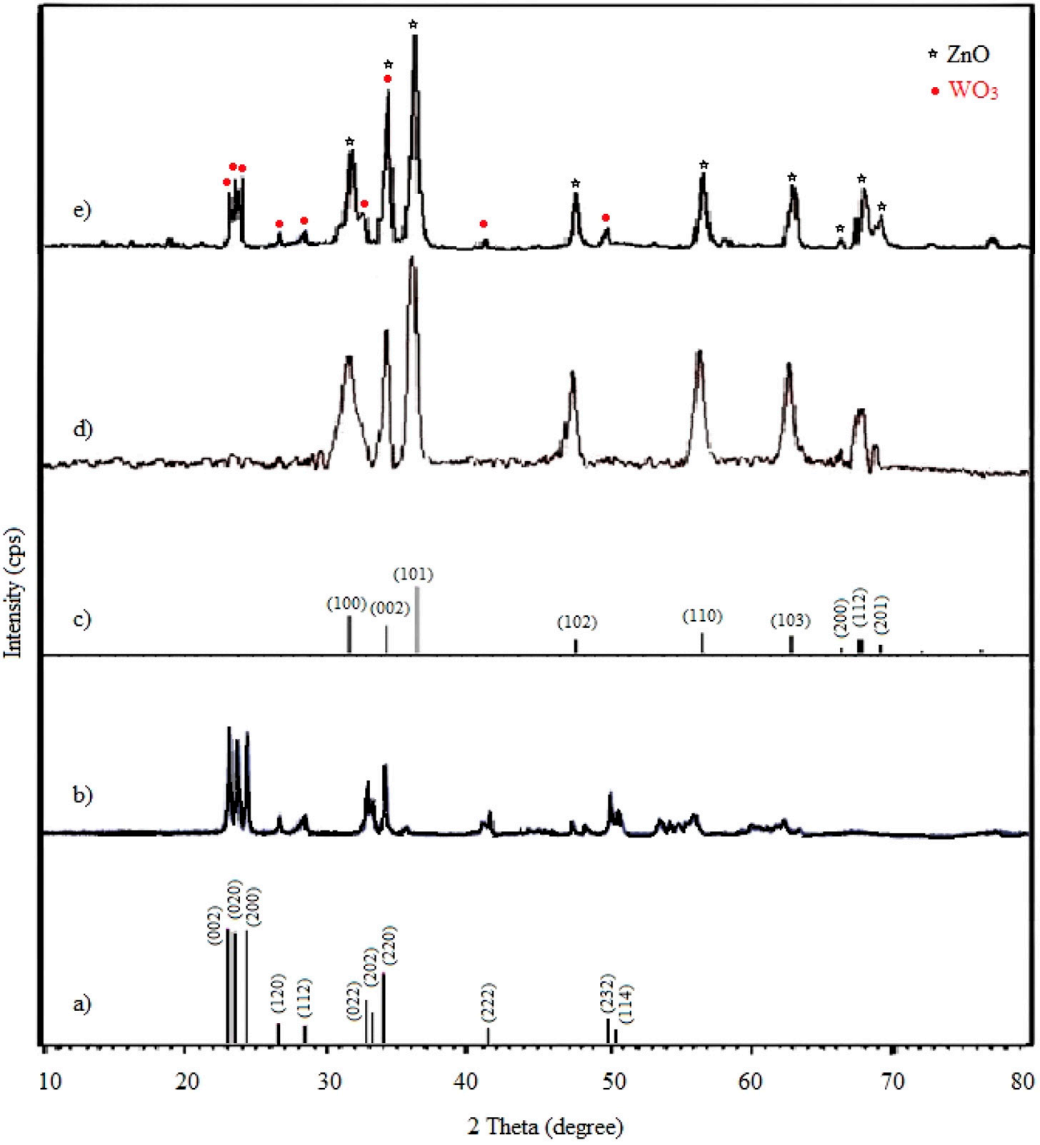
Wide-angle XRD pattern of **(A)** simulated standard WO_3_, **(B)** synthesized WO_3_, **(C)** simulated standard ZnO, **(D)** synthesized ZnO and **(E)** synthesized W-ZnO.

To demonstrate the successful synthesis of NPs and to investigate their crystal phase, XRD measurements have been performed. Figure 2B reveals the monoclinic structure of synthesized WO_3_ by matching it with the standard patterns (JCPDS card no. 83-0950, Figure 2A). Main strong diffraction peaks at 2θ = 23.1°, 23.5°, 24.3°, 26.6°, 28.3°,32.8°, 33.2°, 34.0°, 41.4°, 49.8°, 50,4° are assigned as (002), (020), (200), (120), (112), (022), (202), (220), (222), (232) and (114). The XRD pattern for the synthesized ZnO NPs is shown in Figure 2D. The XRD peaks at 2θ = 31.5°, 34.2°, 36.4°, 47.8°, 56.7°, 62.9°, 66.3°, 67.8° and 69.2° are associated with the (100), (002), (101), (102), (110), (103), (200), (112) and (201) crystalline planes of hexagonal crystal geometry of ZnO, which is associated with JCPSD card no. 01-007-2551 (Figure 2C). In the XRD pattern of W-ZnO (Figure 2E), the diffraction peaks at the 2θ of 23.0°, 23.45°, 24.2°, 26.5°, 28.3°,32.5°, 41.2° and 49.8° corresponded to the (002), (020), (200), (120), (112), (022), (222) and (232) crystal planes of WO_3_. In addition, the diffraction peaks at the 2θ of 31.5°, 34.2°, 36.1°, 47.7°, 56.8°, 63.1°, 66.2°, 68° and 69.1° corresponded to (100), (002), (101), (102), (110), (103), (200), (112) and (201) planes of ZnO, respectively. These results confirmed that W-ZnO was properly synthesized.

**FIGURE 3:**
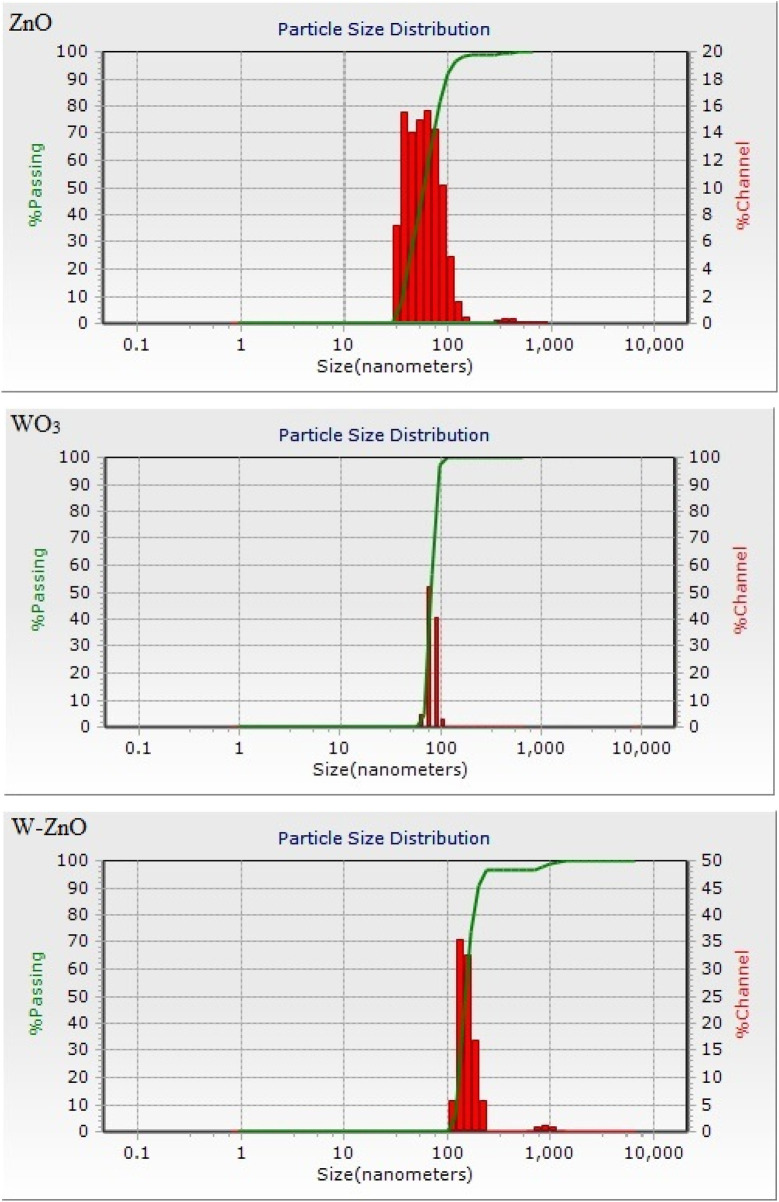
The size distribution charts of ZnO, WO_3_ and W-ZnO particles.

Size is an important factor to describe NPs, hereupon the DLS as a widely used technique for the determination of particle size in colloidal solution was used in this study. Figure 3 shows the particle size distribution graph for the ZnO, WO_3_ and W-ZnO NPs. According to the data obtained from the DLS analysis, the average size of synthesized NPs is 75, 91 and 183 for ZnO, WO_3_ and W-ZnO particles, respectively.

To evaluate the precise and dispersion of measurement values for DLS analysis result, we calculate the standard deviation (*σ*) of the particles. Standard deviation can be calculated using:

$$\sigma =\sqrt{\frac{{\sum f_{i}\left(x_{i}-\bar{x}\right)}^{2}}{n-1}}$$ (8)

where $f_{i}$ , $\bar{x}$ and *n* are the frequency (percent of passing particles), average size and summation of frequencies, respectively. Standard deviations of ZnO, WO3 and W-ZnO NPs were obtained 33, 9 and 17 nm, respectively.

Unlike range and interquartile range, variance is a measure of dispersion that takes into account the spread of all data points in a data set. It’s the measure of dispersion the most often used, along with the standard deviation, which is simply the square root of the variance. The variance is mean squared difference between each data point and the centre of the distribution measured by the mean.

**FIGURE 4:**
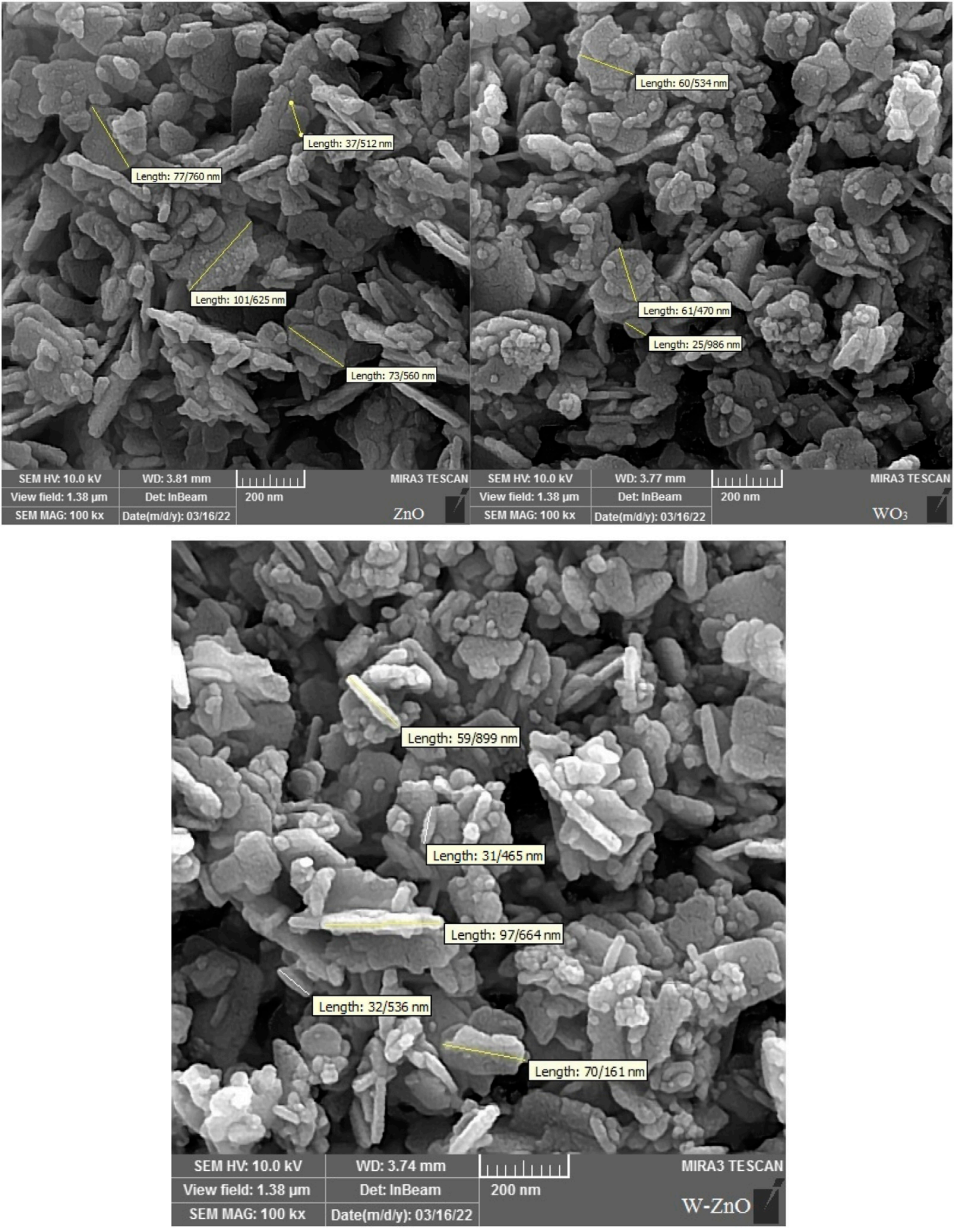
SEM images of ZnO, WO_3_ and W-ZnO.

Scanning electron microscopy (SEM) was used as a powerful technique to describe the morphological characteristics of the synthesized structures (Figure 4). According to the SEM images, it can be concluded that the synthesized W-ZnO has almost a nanosheet framework. By consideration of this figure, it is clear that WO_3_ and ZnO particles have dimensions below 100 nm. It is well recognizable that adding WO_3_ to ZnO did not cause a noticeable change in the particle size and most of the W-ZnO particles have dimensions below 100 nm. In addition, there is approximately no agglomeration in synthesized structures.

**FIGURE 5:**
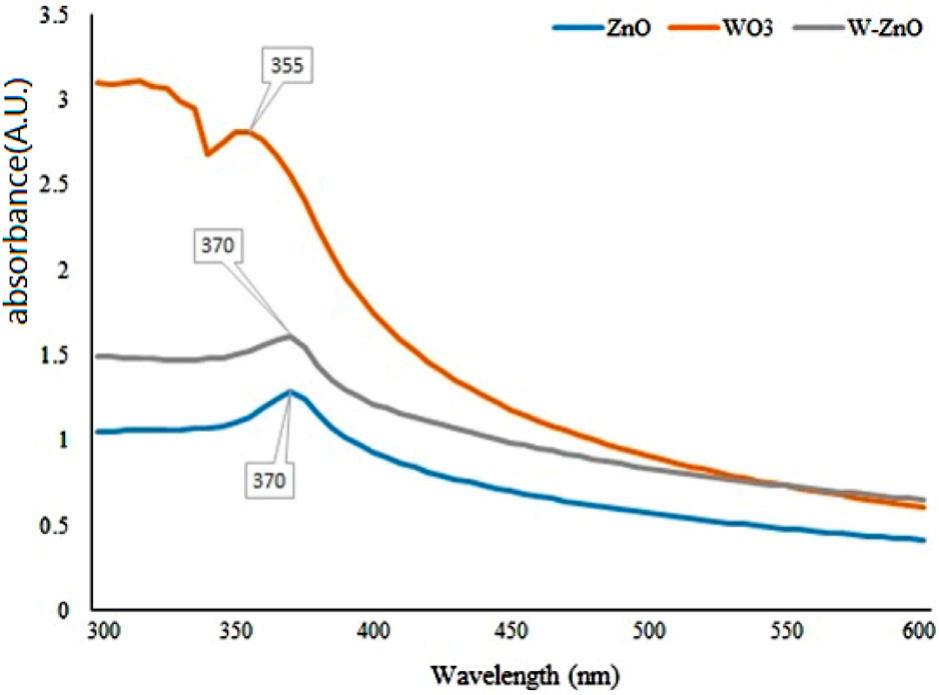
UV–Vis absorption spectra of ZnO, WO_3_ and W-ZnO.

For a more analytical investigation of the synthesized samples, the amount of absorption with the wavelength of 300–600 nm was observed by UV-Vis spectroscopy. As can be seen in Figure 5, the SPR band cantered 370 nm confirms the formation of ZnO NPs (Ghorbani et al., 2015). The excitonic feature of W-ZnO has also appeared at 370 nm. There is a lack of unanimous value for the excitonic point of WO_3_ in the literature (Baishya et al., 2018), However, Figure 5 shows adsorption at 335 nm for WO_3_ which can be consigned to the intrinsic band-gap absorption of WO_3_ (Ramar and Balasubramanian, 2018).

**FIGURE 6:**
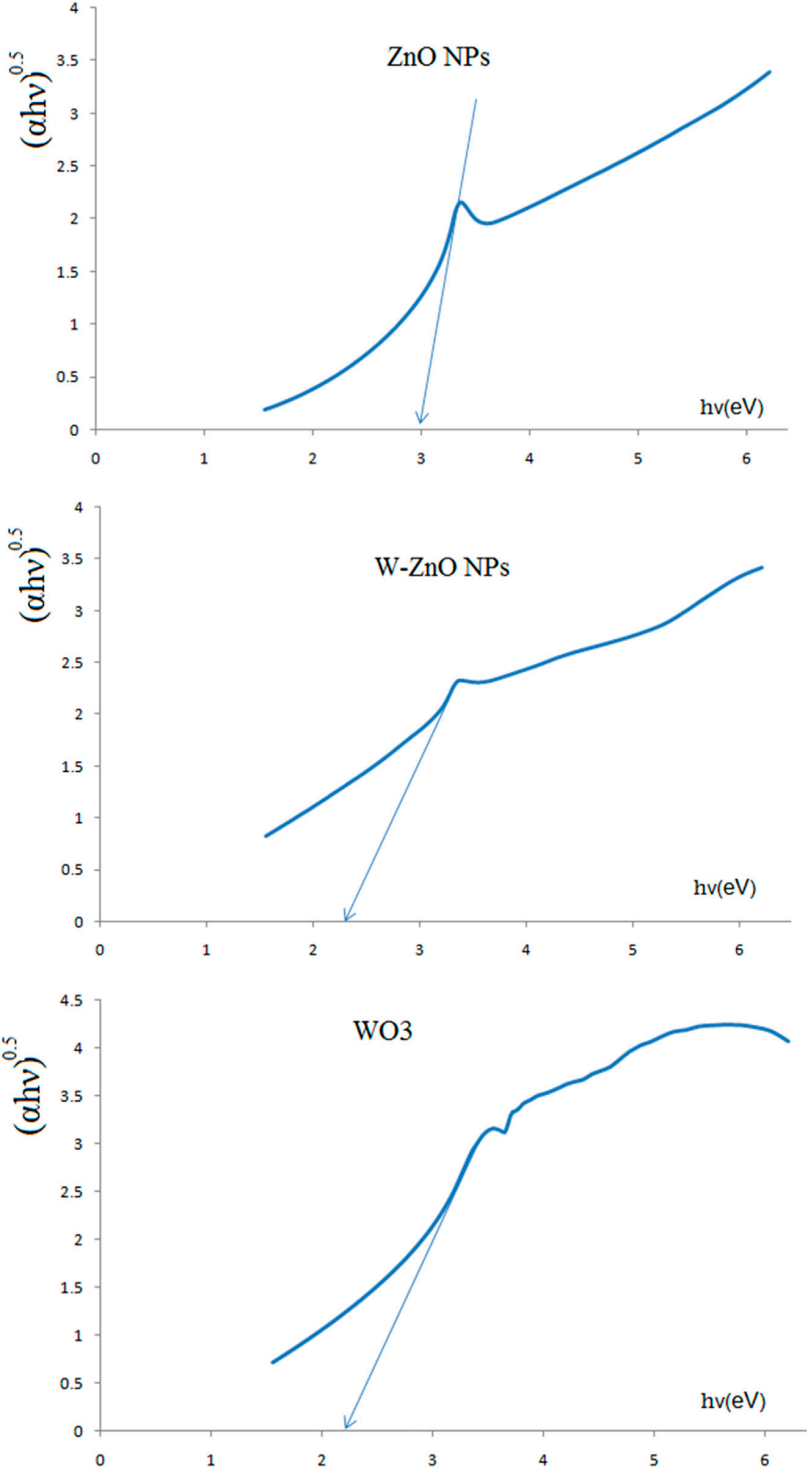
Tauc plots of ZnO, WO_3_ and W-ZnO.

In Figure 6, the colloidal absorption spectrum of ZnO, WO_3_ and W-ZnO NPs in water is plotted in the ultraviolet-visible region. With the help of the Tauc method, the optical energy band gap of NPs can be obtained. In this method, the following relation is used (Eq.3) (Arun et al., 2015).

In Figure 6, the Tauc plot of colloidal ZnO, WO_3_ and W-ZnO NPs in water is plotted. With the help of the Tauc method, the optical energy band gap of NPs can be obtained. In this method, the following relation is used (Arun et al., 2015; Koushki et al., 2019).

$${\left(\alpha h\nu \right)}^{0.5}=A\left(h\nu -E_{g}\right)$$ (9)

where *A* is a constant value, *h* is Planck constant, *ν* is the photon frequency and α is the absorption value. The value on the left side of this equation is plotted against *hν.* By drawing a tangent line to the curve, a bang gap value would be achieved. As can be seen in the table 1 the band gap energy of 3.04 eV was obtained for ZnO NPs and, therefore, it can be considered as semiconductors with relatively high band gap energy, but other NPs have a smaller bandgap (Koushki, 2021), and their higher conductivity can be attributed to the presence of tungsten structures.

**TABLE 1:** Optical coefficients of synthesized NPs, measured using a CW Nd-Yag (green) laser.

Component	_α(1/cm) at 532nm_	$n_{2}$ ( $cm^{2}/W$ )	*β* ( cm/W )	Is (W/cm2)	Band gap energy (*eV*)
ZnO NPs	_0.6_	$-7.5\times 10^{-8}$	$-1.71\times 10^{-3}$	_350_	_3.04_
WO3 NPs	_0.9_	$-1.5\times 10^{-9}$	—	—	_2.2_
W-ZnO NPs	_0.9_	$-9.3\times 10^{-8}$	$-1.46\times 10^{-3}$	_610_	_2.3_

### 5.2 The study of non-linear optical properties of synthesized NPs

**FIGURE 7:**
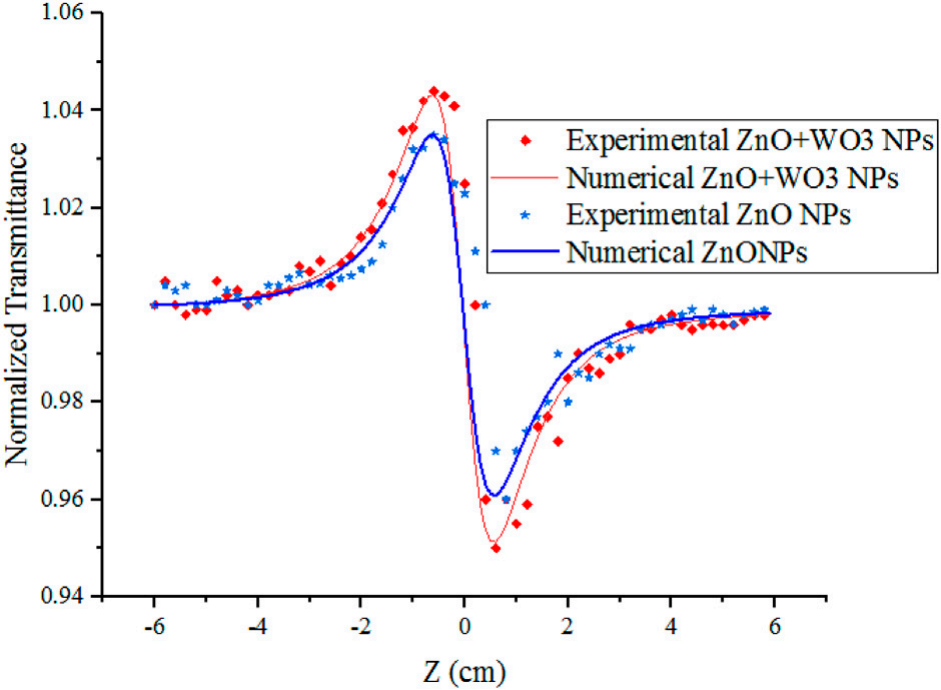
Curves of NPs in close aperture experiment.

The curves of the closed aperture Z-scan measurement of the NPs have been obtained and fitted with Eq. 6. No trustworthy non-linear signal was obtained in the WO_3_ nano colloid according to the measurements made with the Z-scan. For ZnO and W-ZnO NPs, we have the peak-valley distortion according to Figure 7, which indicates the non-linear refractive index $n_{2}$ is negative, as is shown in Table 1. As we know, the negative $n_{2}$ is a result of the thermal lensing effect because, at low laser intensities, non-linearity rather is not related to the electronic transition of energy levels, or at least it can be concluded that the origin of non-linear optical properties is expressed electronically with a very small percentage. In the thermal lensing effect, if we increase the light intensity, it causes the material gets hotter, and we will have a local expansion at the point of light irradiation, so the material will be diluted and the refractive index will decrease (negative in Eq. 1) (Koushki and Majles Ara, 2014). Therefore, the greater the absorption of the material, the more the expansion of the sample, that is, they are directly related to each other. We expect a high refraction change at the wavelength of 532.8 nm because of high absorption in this wavelength (Figure 5). As shown in table 1, there is a direct relation between the non-linear refractive index $n_{2}$ and the absorption coefficient.

**FIGURE 8:**
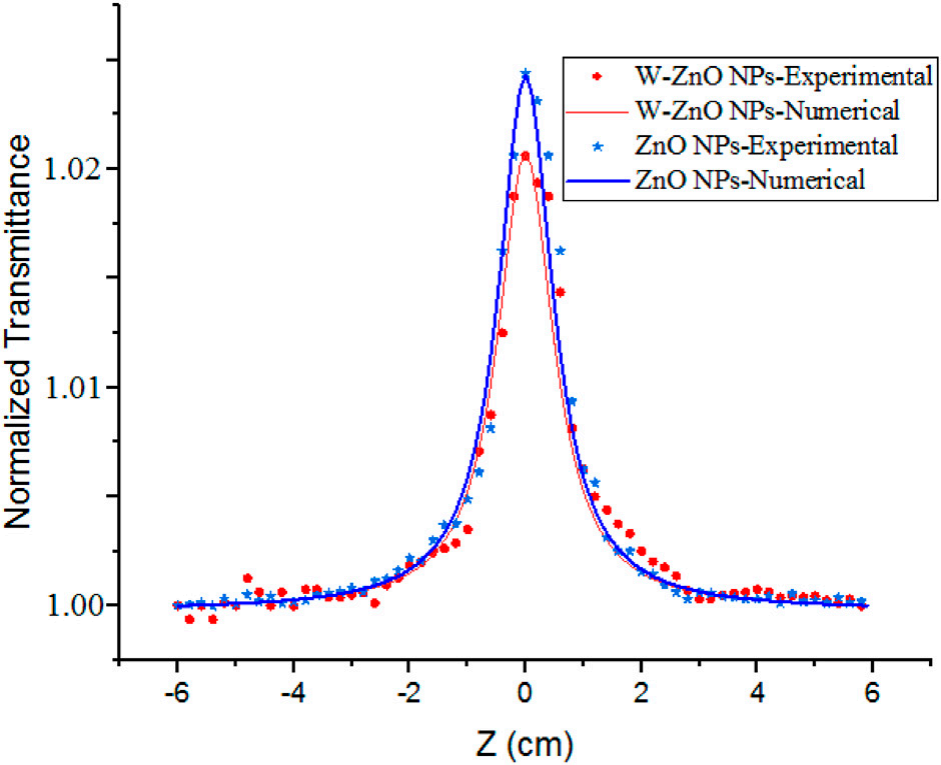
Open aperture curves of ZnO and ZnO-WO_3_NPs.

Figure 8 shows open aperture curves for the synthesized NPs that are plotted with numerical curves (Eq. 7), and non-linear absorption coefficients (*β*) were reported in Table 1 which are negative, and indicate saturation in absorption (SA) is the origin of non-linear absorption. In SA, photons absorbed by NPs enhance the relative population of NPs in exited states and therefore, absorption of new photons experience a decrease, and transmitted power from the particles increases (Purohit, 2020). SA has many applications in the light modulation and laser mode locking. Saturation intensity can be approximately obtained by Is≈−αβ (Sharma et al., 2012; Koushki et al., 2014b), as reported in table 1. Decreasing the saturation intensity for W-ZnO can be related to decreasing the band gap. The smaller the bandgap, the less the probability of electrons staying at a high level, and the creation of population inversion and saturation in absorption will be reduced.

It should be considered that optical non-linearity of metal base nanocolloids and nanocomposites are highly sensitive on the environmental factors around the NPs, and one should not expect completely stable non-linear behavior with the change of these factors. Changes in beam intensity, wavelength, surface agents and dopants can all change the non-linear optical behavior of metal base nanocomposites. The effects of dopants on the change in size of NPs, lattice structure and energy gap and consequently the non-linear optical properties have been proven (Babeela et al., 2019). Also, the effects of surface agents on defect states and morphology can change the amount and even the mechanism type of non-linear response (Sabari Girisun et al., 2018; Monisha et al., 2020). Non-linear optical responses can be tunable and varies with change of beam wavelengths. For example, two photons absorption can be replaced with SA in different wavelengths (Sabari Girisun et al., 2018; Hegde et al., 2019). This response for metal NPs is not only a defect but they can be effective in tunable optical designs such as broadband ultrafast optical limiters.

## 6 Conclusion

In summary, the nanocomposite of zinc tungsten oxide (W-ZnO NPs) was synthesized and characterized using measurement methods such as XRD, DLS, SEM and UV-Vis. Also, the results were compared with pure synthesized ZnO and WO_3_ NPs. Non-linear optical properties of the synthesized composite were measured using the Z-scan technique with a continuous wave 5 mW Nd-Yag laser beam. A negative non-linear absorption coefficient of the components was obtained, and it shows that saturation absorption occurred in this composite. In comparison with pure ZnO NPs, non-linear absorption decreases that can be attributed to the negligible optical response of WO_3_ structures. Also, the negative value of the close aperture Z-scan curve shows that the thermal lensing effect is the main reason for the third-order non-linear refraction.

## Data Availability

The raw data supporting the conclusion of this article will be made available by the authors, without undue reservation.
